# Outcomes in Patients With Pulmonary Embolism Treated With Mechanical Thrombectomy or Anticoagulation Alone

**DOI:** 10.1016/j.jscai.2025.102611

**Published:** 2025-05-01

**Authors:** Hassan Saleh, Ibrahim Khaleel, Connor Kerndt, Paul Weber, Matthew Hollowell, Connor McCalmon, Theresa Pasion, Mohammad Ahmed, Mazin Habhab, Nolan Rossman, Jessi Parker, Brian Trethowan, Marcel Letourneau

**Affiliations:** a Division of Cardiology, Department of Heart and Vascular Medicine, Corewell Health West and Michigan State University, Grand Rapids, Michigan; b Michigan State University College of Human Medicine, Grand Rapids, Michigan; c Division of Hospitalist Medicine, Department of Internal Medicine, Corewell Health West and Michigan State University, Grand Rapids, Michigan; d The Office of Research and Education, Scholarly Activity and Scientific Support at Corewell Health West, Grand Rapids, Michigan; e Division of Cardiothoracic Critical Care, Heart and Vascular Medicine, Corewell Health West and Michigan State University, Grand Rapids, Michigan

**Keywords:** anticoagulation, catheter-directed therapy, pulmonary embolism, pulmonary embolism severity index, thrombectomy

## Abstract

**Background:**

Pulmonary embolism (PE) is a leading cause of cardiovascular death; little data exist on whether mechanical thrombectomy confers a mortality benefit. Using a retrospective review, 311 consecutive patients with PE who underwent aspiration thrombectomy were compared to 309 propensity score–matched patients with PE treated with anticoagulation alone.

**Methods:**

Using a retrospective review, we identified 311 consecutive patients with PE who underwent mechanical thrombectomy along with standard of care; we then identified 1841 patients admitted with a primary diagnosis of PE and used propensity score matching to identify 309 patients with similar pulmonary embolism severity index (PESI) scores and variables. We then evaluated 2-year outcomes between the 2 groups.

**Results:**

Of the 311 patients treated with thrombectomy, 262 were at elevated risk by the European Society of Cardiology (ESC) stratification, 261 had a positive simplified pulmonary embolism severity index (sPESI) and 208 were of PESI class III or higher. Of the 309 patients treated with anticoagulation alone, 261 had elevated risk by ESC stratification, 257 had a positive sPESI, and 201 were PESI class III or higher. When all patients were evaluated, there was a mortality benefit starting at 30 days in patients undergoing thrombectomy; when patients with metastatic cancer were excluded, the mortality benefit was only seen in higher-risk patients. Low-risk patients with or without right ventricular strain had similar mortality whether managed with thrombectomy or anticoagulation alone, with numerically more significant bleeding, stroke, and recurrent pulmonary emboli.

**Conclusions:**

In this single-center, retrospective review, patients with PE who were of ESC high risk and who underwent aspiration thrombectomy with a FlowTriever System (Inari Medical) had a statistically significant reduction in mortality compared to a propensity score–matched group treated with anticoagulation alone; separation in mortality curves continued at 2 years. Our findings also suggest that low-risk patients perform equally well with or without thrombectomy but incur numerically more bleeding events, stroke, and recurrent pulmonary emboli.

## Introduction

Pulmonary embolism (PE) remains the third leading cause of cardiovascular death in the United States.^1^ Mortality from PE can be estimated using risk calculators that have identified factors that correlate with 30-day mortality including cancer, congestive heart failure, chronic obstructive pulmonary disease, hypotension, and tachypnea.^1^, ^2^, ^3^ Until recently the mainstay for treating PE has been anticoagulation alone with thrombolytics reserved as a last-line therapeutic.^4^^,^^5^ Evidence for thrombolytics in massive PE is limited, whereas, in intermediate-risk PE, thrombolytics improve hemodynamics but not mortality, with a significantly higher rate of bleeding and hemorrhagic stroke.^6^

Although catheter-directed therapies (CDT) have existed, trial outcomes evaluating their efficacy have focused on clinical parameters rather than mortality. As of date, no randomized trials have been published comparing anticoagulation alone to CDT along with anticoagulation.^7^, ^8^, ^9^, ^10^, ^11^ Results of trials are pending; the HI-PEITHO trial which is randomizing intermediate-high risk PE patients to EkoSonic Endovascular System (EKOS) versus anticoagulation alone is still enrolling. The PE-TRACT trial is randomizing patients with PE and RV: LV (right ventricle: left ventricle) ratio >1 on computed tomography (CT) to anticoagulation alone or to CDT along with anticoagulation. Study end points include differences in costs and functional capacity among others with results expected in 2028. Results of the PEERLESS II trial which randomized intermediate-risk PE patients to mechanical thrombectomy using the FlowTriever System (Inari Medical) or to alternative CDT did not demonstrate a mortality benefit.^9^^,^^12^^,^^13^

Given mechanical thrombectomy does carry procedural risk, with unknown impact on medical costs, lengths of stay, and mortality, we evaluated these criteria in 311 consecutive patients who underwent mechanical thrombectomy and compared them to 309 propensity score–matched (PSM) patients who were treated with anticoagulation alone. We observed the 2-year mortality, length of stay, major bleeding as well as rates of stroke (hemorrhagic or ischemic).

## Methods

### Study design

The study was a single quaternary center, retrospective review, assessing 311 patients with PE treated with mechanical thrombectomy using the FlowTriever System (Inari Medical) along with anticoagulation and compared outcomes to 309 PSM patients treated with anticoagulation alone between July 1, 2019, and April 1, 2022. Propensity score matching was performed using pulmonary embolism severity index (PESI) variables. Patients in the thrombectomy group had their procedure at any time during their hospitalization. Patients in the PSM group had a primary diagnosis of PE and were treated with anticoagulation at any time during their hospitalization. Patient admission characteristics were used to derive PESI and simplified pulmonary embolism severity index (sPESI) scores.

Results of CT, transthoracic echocardiogram, troponin assay, and NT-proBNP along with thrombectomy procedural reports were reviewed. RV strain was present on CT if the RV: LV ratio was >1, or if flattening of the septum was reported. RV strain on transthoracic echocardiogram was present if there was systolic flattening of the septum, the TAPSE was <17 mm, S’ <9.5 cm/s, or if the RV fractional area of change was <35%. Positive cardiac biomarkers were defined as a high sensitivity troponin level of >14 ng/L in women and >22 ng/L in men, or a NT-proBNP level >400 pg/mL. Propensity score matching for the control group was based on PESI variables (Tables 1 and 2). Institutional review board approval was obtained prior to initiating the study.

**Table 1:** Pulmonary embolism severity index criteria.

Variable	Thrombectomy	Propensity score-matched group	*P* value
PESI score	99 (79-126)	101 (78-127)	.96
Age, y	64 (51-73)	64 (50-75)	.76
Male sex	172 (55.3)	168 (54.4)	.81
History of non–skin cancer	58 (18.7)	70 (23)	.81
History of heart failure	21 (6.8)	23 (7.4)	.74
History of lung disease	60 (19.3)	64 (20.7)	.66
Altered mental state	21 (6.8)	17 (5.5)	.52
Heart rate	105.9 ± 20.7	105.6 ± 20.3	.87
Initial heart rate ≥110 bpm	136 (43.7)	132 (42.7)	.80
Systolic blood pressure, mm Hg	132.7 (112-152)	111 (103-122)	<.0001
Systolic blood pressure <100 mm Hg	45 (14.5)	42 (13.6)	.73
Respiratory rate, breath/min	21 (18-26)	23 (20-27)	<.0001
Respiratory rate ≥30/min	37 (11.9)	45 (14.6)	.33
Temperature (°F)	98.1 (97.7-98.4)	97.7 (97.3-97.9)	<.0001
Temperature <96.8 °F (36 °C)	8 (2.6)	10 (3.2)	.62
Oxygen saturation, %	90.5 (88-95)	90.5 (88-95)	–
Oxygen saturation <90%	156 (50.2)	154 (49.8)	.94

Values are median (IQR) or n (%).

PESI, pulmonary embolism severity index.

**Table 2:** Cohort characteristics before and after propensity score matching.

Variable	Before propensity score matching			After propensity score matching		
	Anticoagulation group (n = 1841)	Thrombectomy (n = 311)	*P* value	Anticoagulation group (n = 309)	Thrombectomy (n = 311)	*P* value
Age, y	64 (50-75)	64 (51-73)	.9242	64 (50-75)	64 (51-73)	.76
Male sex	933 (50.7)	172 (55.3)	.1311	168 (54.4)	172 (55.3)	.81
History of cancer	446 (24.3)	58 (18.7)	.0317	70 (23)	58 (18.7)	.81
History of heart failure	267 (14.5)	21 (6.8)	.0002	23 (7.4)	21 (6.8)	.75
History of lung disease	583 (31.7)	60 (19.3)	<.0001	64 (20.7)	60 (19.3)	.66
Altered mental state	219 (11.9)	21 (6.8)	.0077	17 (5.5)	21 (6.8)	.52
Heart rate ≥110 bpm	561 (30.5)	136 (43.7)	<.0001	132 (42.7)	136 (43.7)	.80
SBP <100 mm Hg	451 (24.5)	45 (14.5)	.0001	42 (13.6)	45 (14.5)	.73
Respiratory rate ≥30/min	203 (11.0)	37 (11.9)	.6519	45 (14.6)	37 (11.9)	.33
Temperature <98.6 °F	50 (2.7)	8 (2.6)	.8850	10 (3.2)	8 (2.6)	.62
Oxygen saturation <90%	415 (22.6)	156 (50.2)	<.0001	154 (49.5)	156 (50.2)	.94

Values are median (IQR) or n (%).

SBP, systolic blood pressure.

### Inclusion and exclusion criteria

Patients in the thrombectomy group were hospitalized patients aged 18 years or older, who had a diagnosis of PE by CT, and underwent mechanical thrombectomy during their hospitalization. All patients aged 18 years or older who underwent thrombectomy between July 1, 2019, and April 1, 2022 were included in this analysis.

Patients in the PSM group were included if they were aged 18 years or older and hospitalized between July 1, 2019, and April 1, 2022, with a primary diagnosis of PE diagnosed by CT or ventilation-perfusion imaging and treated with anticoagulation.

### Study outcomes

Outcomes assessed were in-hospital death, 30-day, 1-year, and 2-year mortality rates, major bleeding defined by requirement for blood transfusion, length of stay, and rate of stroke hemorrhagic or embolic. Recurrent PE rates at 2 years are reported for low-risk patients.

### Statistical analysis

Baseline variables to derive PESI and sPESI scores were collected and used to derive sPESI and PESI cores. For patients undergoing thrombectomy, any systolic blood pressure of <100 mm Hg, heart rate of ≥110 bpm, respiratory rate ≥30/min, or oxygen saturation of <90% that was documented prior to thrombectomy was used when calculating the PESI and sPESI scores.

Normal numeric data are expressed as mean ± SD and analyzed using the 2-same independent *t* test, not normal numeric data are expressed as median (25th, 75th percentile) and analyzed using Wilcoxon rank sum. Categorical variables are expressed as frequent (percentage) and analyzed using the χ^2^ test. Propensity score matching was performed using 1:1 greedy caliper matching using random matching with a caliper width of 0.40. Matching was based on PESI variables (Tables 1 and 2). One thousand eight hundred forty-one anticoagulation alone patients were identified who met the inclusion criteria and who then underwent propensity score matching, resulting in 309 matched patients. Kaplan-Meier curves were generated for analysis of time to mortality. Standard differences were calculated before and after propensity score matching to evaluate the effect of matching. Statistical analyses were performed using Statistical Analysis System version 9.4.

## Results

### Baseline characteristics and comparability of groups

Using the previously mentioned inclusion criteria, 311 consecutive patients diagnosed with PE and treated with thrombectomy were identified. One thousand eight hundred forty-one patients hospitalized with PE who were treated with anticoagulation alone were identified and using PESI variables, 309 patients were propensity score matched for comparison. Baseline characteristics and PESI variables are provided (Table 1, Table 2, Table 3).

**Table 3:** Baseline characteristics.

	Thrombectomy group	Propensity score–matched group	*P* value
Age, y			
Median	64 (51-73)	64 (50-75)	.76
Mean	62.2 ± 15.2	62.15 ± 17.2	.97
<65	161 (51.8)	155 (50.2)	.34
65-75	92 (29.6)	83 (26.9)	.29
>75	58 (18.6)	71 (22.9)	.91
Male sex	172 (55.3)	168 (54.4)	.81
Race			
White	279 (89.7)	267 (86.4)	.15
Black	21 (6.8)	33 (10.7)	.96
Other	11 (3.5)	9 (2.9)	.33
Body mass index			
<18.5 kg/m^2^	1 (0.3)	6 (1.9)	.97
18.5-24.9 kg/m^2^	36 (11.6)	39 (12.6)	.66
25-29.9 kg/m^2^	74 (23.8)	101 (32.7)	.002
>30 kg/m^2^	200 (64.3)	163 (52.8)	.002
eGFR, mL/min/1.73 m^2^			
≥60	260 (83.6)	243 (78.6)	.056
45 to <60	32 (10.3)	39 (12.6)	.82
30 to 44	17 (5.5)	21 (6.8)	.76
15 to 29	2 (0.6)	5 (1.6)	.87
<15	0 (0)	1 (0.3)	.84
Smoking status			
Never	171 (55)	163 (52.8)	.29
Previous	108 (34.7)	109 (35.2)	.56
Current	32 (10.3)	37 (12)	.75
Use of thrombolytics	8 (2.6)	11 (3.6)	.76
Cardiac arrest	7 (2.3)	9 (3.3)	.70
Preprocedure vasopressor	23 (7.4)	10 (3.2)	.01
COVID-19 positive	54 (17.4)	26 (8.4)	.0004
Metastatic cancer	25 (8)	45 (14.5)	.005
Right ventricular strain, CT^a^	252 (81)	93 (31.1)	<.0001
Right ventricular dysfunction, TTE^b^	172 (55.3)	75 (33.4)	<.0001
Elevated NT-proBNP or troponin^c^	274 (88.1)	101 (32.7)	<.0001
CT for diagnosis	311 (100)	299 (96.8)	.0007
Ventilation perfusion for diagnosis	0 (0)	10 (3.2)	.0009

Values are n (%) unless otherwise noted. Given the incomplete data for the PSM group for certain testing, percentages reflect the number of patients with data available.

CT, computed tomography; eGFR, estimated glomerular filtration rate; PSM, propensity score–matched; TTE, transthoracic echocardiogram.

a Represents 299 PSM patients who had CT results.

b Represents 230 patients who had TTE results.

c Represents 302 PSM patients who had either NT-proBNP or troponin assessed.

Pulmonary embolism severity index variables between the groups were well matched. All PESI variables were similar with no significant differences. The median PESI score was 99 (79-126) in the thrombectomy group and 101 (78-127) in the PSM group, both corresponding to PESI class III. The number of patients in each PESI class was also similar between the 2 groups.

The thrombectomy and PSM groups were compared to the groups from the sPESI and PESI derivation studies (Table 4). Compared to the derivation studies, our population had a higher incidence of the male sex, chronic renal disease, HR >110 bpm, oxygen saturation <90%, and a lower occurrence of hypothermia. The differences in vitals may suggest more comorbid conditions in our patients, or be a function of more data points available for analysis when using an electronic medical record in the 2020s compared to the documentation available in the 2010s when the sPESI and PESI studies were performed.

**Table 4:** Percentage of patients with PESI variable between cohorts.

Characteristic	Thrombectomy (n = 311)	PSM group (n = 309)	PESI derivation (n = 10,354)	sPESI derivation (n = 995)
Male sex	55.3	54.4	39.6	45.1
Cancer	18.3	23	19.9	24
Heart failure	6.8	7.4	16.1	6.9
Chronic lung disease	19.3	20.7	18.2	7.4
Chronic renal disease	16.4	21.4	4.4	–
Temperature <36 °C	2.6	3.2	16.7	9.5
Pulse >110/min	43.7	42.7	29.2	18.4
Systolic blood pressure <100 mm Hg	14.5	13.6	10.6	8.9
Respiratory rate >30/min	11.9	14.6	14.5	6.8
Altered mental status	6.8	5.5	6.9	0.2
Oxygen saturation <90%	50.2	49.8	8	25.8

Values are %.

PESI, pulmonary embolism severity index; sPESI, simplified pulmonary embolism severity index; PSM, propensity score–matched.

Non-PESI variables are also reported. The thrombectomy group had a higher proportion of patients with BMI >30 kg/m^2^ (54.3% vs 52.8%; *P* = .0016). The rate and stages of chronic kidney disease, smoking status, use of thrombolytics, and rate of cardiac arrest were all similar.

The thrombectomy group did have a higher rate of preprocedural vasopressor use, COVID positivity within 30 days of hospitalization, right ventricular strain, and a higher number of patients with positive cardiac biomarkers. Although the rates of cancer were similar between the groups (23% vs 18.7%; *P* = .8076), the rate of metastatic disease was significantly higher in the PSM group (14.5% vs 8%; *P* = .0099).

Despite propensity score matching, the thrombectomy group had a higher acuity when stratifying by the European Society of Cardiology (ESC) risk model. In the thrombectomy group, there were 39 high-risk patients, 181 intermediate high-risk, and 42 intermediate low-risk patients whereas in the PSM anticoagulation group, there were 23 high-risk patients, 59 intermediate high-risk, and 179 intermediate low-risk patients. The difference was driven by an increased rate of RV strain on CT (81%) and positive cardiac biomarkers (88.1%) in the thrombectomy group, compared to the PSM group which had rates of 31.1% and 32.7%, respectively. This was also partially due to less testing in the PSM group, in which 7 patients had neither a troponin nor NT-proBNP measured and 81 had only 1 of these measured.

Notably, the PSM group had similar mortality rates as seen in published numbers from the International Cooperative Pulmonary Embolism Registry.^14^ The registry reported 90-day mortality rates for massive and nonmassive PE as 52.4% and 14.7%, respectively, while patients in the PSM anticoagulation group had 90-day mortality rates of 55% and 11.7%, respectively.

### Short term mortality

There were 14 (4.5%) mortalities in the thrombectomy group and 17 (5.4%) in the PSM group during hospitalization, which was not significantly different (difference, –0.9%; *P* = .28; relative risk [RR], 0.82; 95% CI, 0.41-1.63).

There were 15 (4.8%) mortalities in the thrombectomy group and 28 (9.1%) in the PSM group at 30 days, which was significantly different (difference, –4.3%; *P* = .02; RR, 0.53; 95% CI, 0.29-0.98).

### One and 2 year mortality

At 1 year, there were 40 (12.9%) mortalities in the thrombectomy group and 65 (21%) in the PSM anticoagulation group, which was significantly different (difference, –8.1%; *P* = .003; RR, 0.61; 95% CI, 0.43-0.88).

At 2 years, there were 50 (16.1%) mortalities in the thrombectomy group and 79 (25.6%) in the PSM anticoagulation group, which was significantly different (difference, –9.5%; *P* = .002; RR, 0.63; 95% CI, 0.46-0.86).

### ESC high-risk

Using the ESC prognostic model, in the thrombectomy group, there were 39 high-risk patients and in the PSM anticoagulation group, there were 23 high-risk patients.

At 30 days, there were 6 (15.4%) deaths among the 39 thrombectomy patients and 12 (52.2%) deaths among the 23 PSM anticoagulation patients (difference –36.8%; *P* = .004; RR, 0.29; 95% CI, 0.13-0.68).

At 1 year, there were 13 (33.3%) deaths among the 39 thrombectomy patients and 14 (60.8%) deaths among the 23 PSM anticoagulation patients (difference, –27.5%; *P* = .03; RR, 0.55; 95% CI, 0.32-0.95).

At 2 years there were 15 (38.5%) deaths among the thrombectomy patients and 17 (73.9%) deaths among the 23 PSM anticoagulation patients (difference, –35.4%; *P* value = .006; RR, 0.54; 95% CI, 0.33-0.83).

### When metastatic cancer is excluded

#### sPESI positive or PESI class III+ without metastatic cancer

In the thrombectomy group, there were 237 patients without metastatic cancer who were sPESI positive or had a PESI classification of III or higher; there were 10 mortalities at 30 days (4.2%), 25 at 1 year (10.5%), and 32 at 2 years (13.5%).

In the PSM anticoagulation group, there were 215 patients without metastatic cancer who were sPESI positive or had a PESI classification of III or higher; there were 13 mortalities at 30 days (6%), 29 at 1 year (13.5%), and 41 at 2 years (19.1%).

The difference in mortality at 30 days, 1 year, and 2 years was not significantly different between the groups.

### ESC intermediate high-risk or high-risk without metastatic cancer

In the thrombectomy group, there were 203 patients without metastatic disease who were ESC intermediate high-risk or high-risk, with 8 mortalities at 30 days (3.9%), 21 at 1 year (10.3%), and 27 at 2 years (13.3%).

In the PSM anticoagulation group, there were 76 patients without metastatic disease who were ESC intermediate high-risk or high-risk, with 14 mortalities at 30 days (18.4%), 21 at 1 year (27.6%), and 26 at 2 years (34.2%).

The absolute difference in mortality between groups at 30 days, 1 year, and 2 years was –14.5%, *P* value .0009, –17.3%, *P* value .0009 and –20.9%, *P* value .0002, respectively, all of which were significantly different.

### Lower-risk patients

#### PESI class I or II

In the thrombectomy group, there were 103 patients who were classified into PESI class I or II, with 1 morality at 30 days (0.97%), 4 mortalities at 1 year (3.8%), and 5 mortalities at 2 years (4.8%). Of the 103 patients, there were 6 patients with recurrent PE within 2 years (5.8%).

In the PSM anticoagulation group, there were 108 patients who were classified into PESI class I or II, with 2 mortalities at 30 days (1.8%), 2 mortalities at 1 year (1.8%), and 5 mortalities at 2 years (4.6%). Of the 108 patients, there were 3 patients with recurrent PE within 2 years (2.8%).

The absolute difference in mortality between groups at 30 days, 1 year, and 2 years was –0.83%, 2%, and 0.2%, respectively, all of which are not significantly different.

The absolute difference in recurrent PE at 2 years was 3%, which was not significantly different.

### PESI class I or II with RV strain and or positive biomarkers

In the thrombectomy group, there were 100 patients with right ventricular strain or positive cardiac biomarkers. In the 100 patients, the mortality was 1 at 30 days (1%), 4 at 1 year (4%), and 6 at 2 years (6%). Eighty-six of the 100 patients had both right ventricular strain and positive cardiac biomarkers; the mortality was 1 at 30 days (1.7%), 4 at 1 year (4.7%), and 6 at 2 years (7%).

In the PSM anticoagulation group, there were 41 patients with right ventricular strain or positive cardiac biomarkers. In the 41 patients, the mortality was 0 at 30 days (0%), 2 at 1 year (4.8%), and 3 at 2 years (7.3%). Sixteen of the 41 patients had both right ventricular strain and positive cardiac biomarkers; there was no mortality at 30 days (0%) and 1 year (0%), and the mortality was 1 at 2 years (6.3%).

The absolute difference in mortality between groups at 30 days, 1 year, and 2 years was 1%, –0.8%, and –1.3, respectively, all of which are not significantly different.

### Adverse events and length of stay

**Figure 1:**
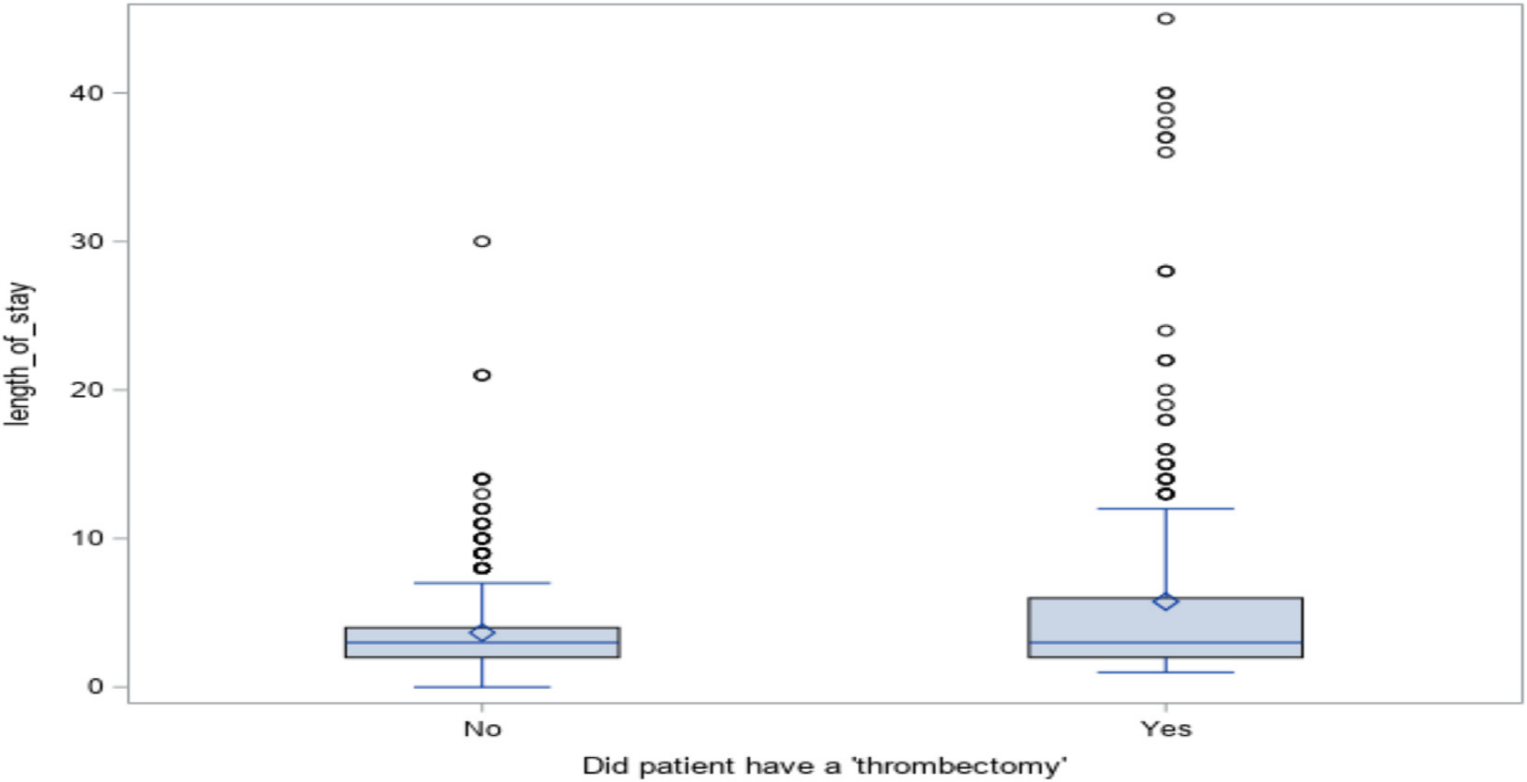
**Distribution of length of stay between thrombectomy and propensity score–matched group**.

The rate of adverse events and median length of stay were similar in both groups. The thrombectomy group had a significantly wider distribution in length of stay (Table 5, Figure 1).

**Table 5:** Adverse events and length of stay.

	Thrombectomy	Propensity score–matched group	*P* value
Length of stay, d	3 (2-6)	3 (2-4)	<.0001
Transfusion	22 (7.1)	15 (4.9)	.12
Stroke	12 (3.9)	8 (2.6)	.19

Values are median (IQR) or n (%).

In the thrombectomy group, 22 (7.1%) patients had a blood transfusion compared to 15 (4.9%) patients in the PSM anticoagulation group. Stroke, either hemorrhagic or embolic, occurred in 12 (3.9%) patients in the thrombectomy group, and in 8 (2.6%) patients in the PSM anticoagulation group (Table 5, Figure 1).

## Discussion

**Figure 2:**
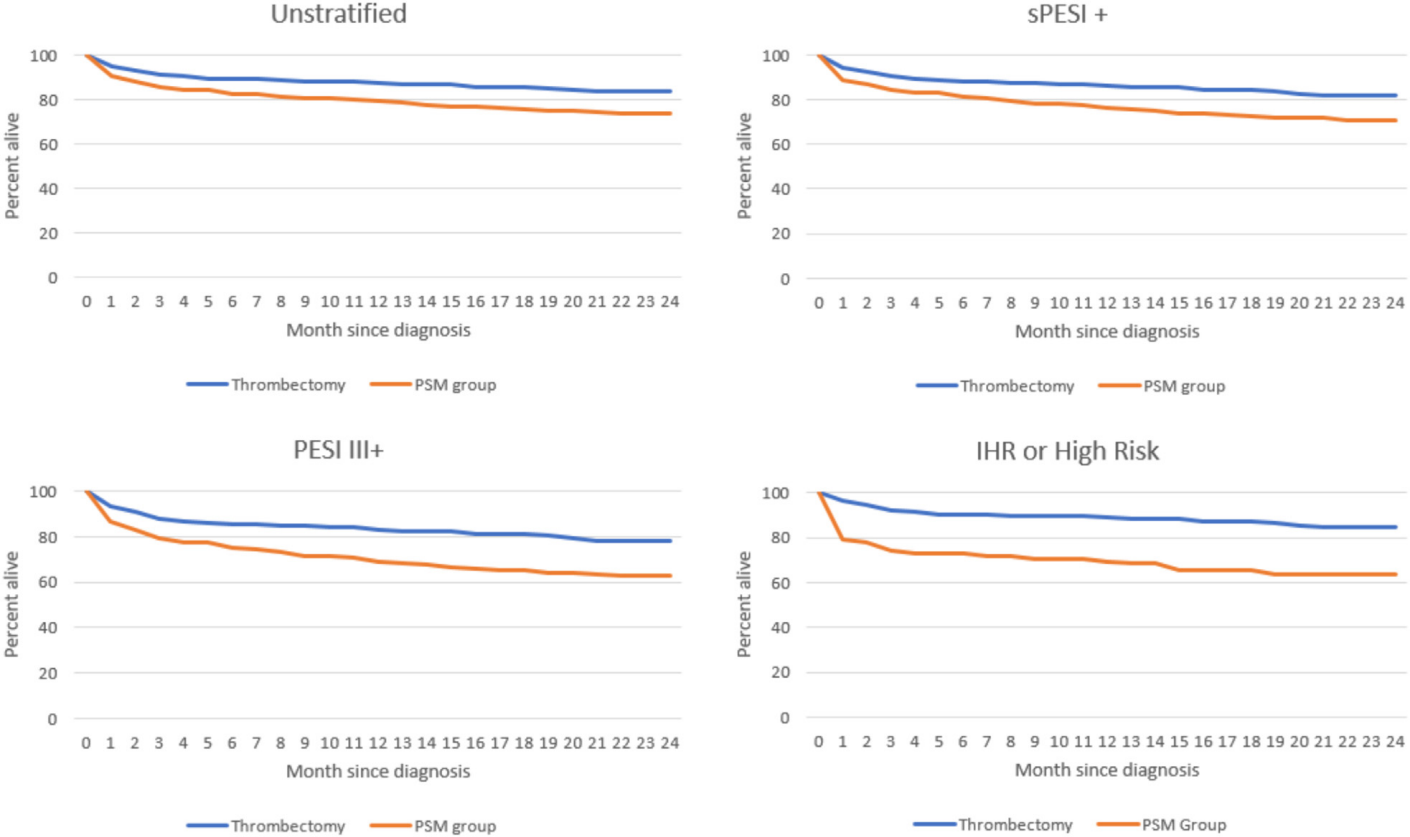
**Survival curves using select stratification methods.** IHR, intermediate-high risk; PESI, pulmonary embolism severity index; sPESI, simplified pulmonary embolism index; PSM, propensity score–matched.

In this PSM, retrospective cohort study, we examined adverse events and 2-year mortality in 311 consecutive patients with PE treated with mechanical thrombectomy and 309 PSM patients treated with anticoagulation alone. Although CDT for PE have become widely adopted, a morality benefit has yet to be demonstrated.^9^, ^10^, ^11^^,^^13^ This review is the first to compare outcomes in patients treated with thrombectomy and anticoagulation or anticoagulation alone. The results suggest that in patients with high-risk and likely intermediate high-risk PE, there is a mortality benefit from mechanical thrombectomy, whereas in those at lower risk, there is not (Figure 2 and Central Illustration).

**Central Illustration:**
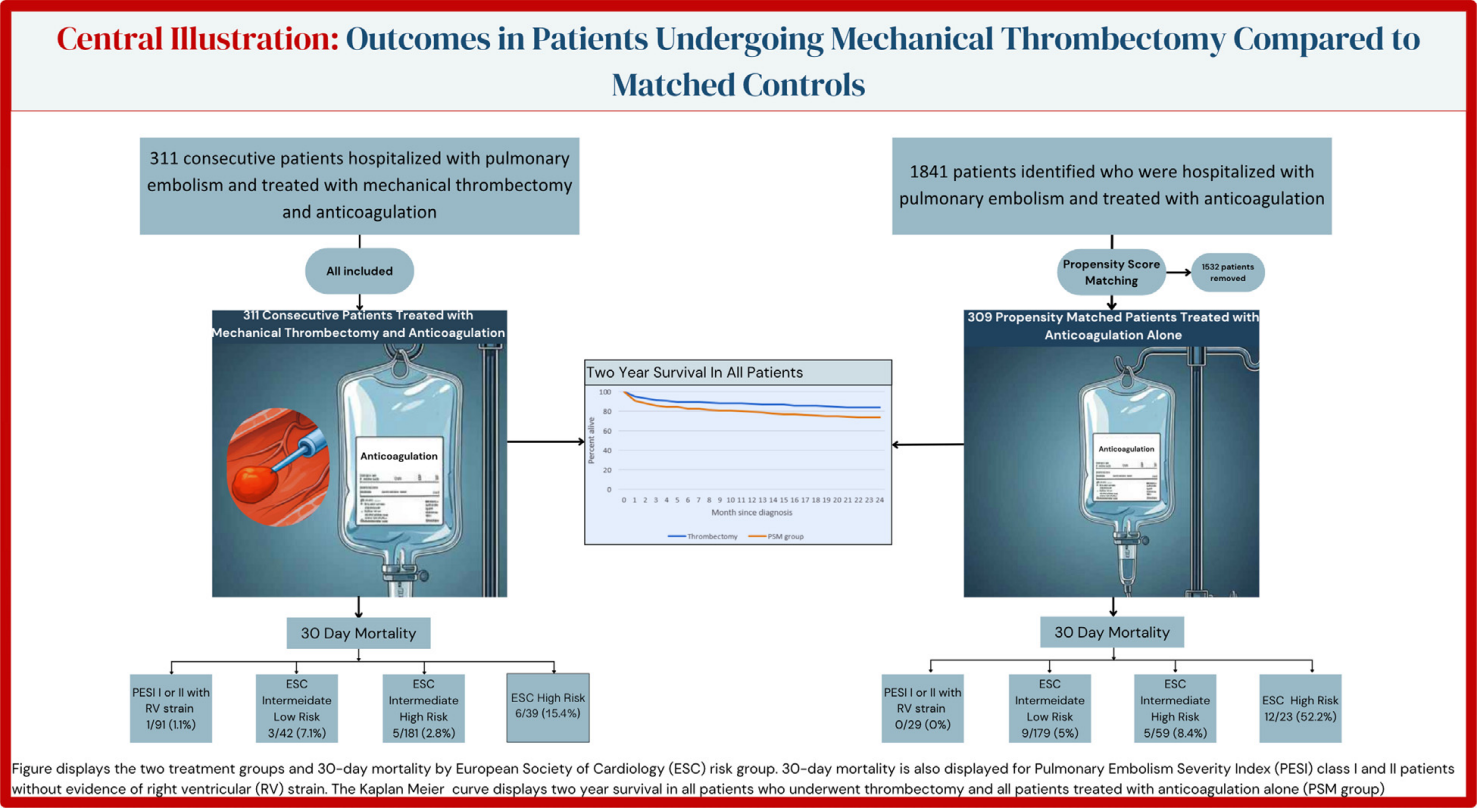
The figure displays the 2 treatment groups and 30-day mortality by the European Society of Cardiology (ESC) risk group. Thirty-day mortality is also displayed for pulmonary embolism severity index (PESI) class I and II patients without evidence of right ventricular (RV) strain. The Kaplan-Meier curve displays the 2-year survival in all patients who underwent thrombectomy and all patients treated with anticoagulation alone (propensity score–matched [PSM] group).

Given the key objective of this retrospective review was to assess adverse events and mortality in patients undergoing thrombectomy, propensity score matching was used to identify comparable patients treated with anticoagulation alone. Propensity score matching has become a popular statistical method employed in retrospective studies; briefly, propensity score matching is a statistical method that estimates the conditional probability of an exposure given specified confounders and is used to address these confounders.^15^^,^^16^^,^^17^ We choose to use the PESI scoring for propensity score matching given its validation and endorsement by both ESC and ACC guidelines. PESI scoring was consistently applied across patients.^4^^,^^5^

This was a limitation of the study as using PESI variables for propensity score matching resulted in the thrombectomy group having significantly more patients with RV strain on CT. As a measure to evaluate this, patients were also stratified by ESC classification (Table 6) to compare mortality. Notably, as mentioned elsewhere, the PSM group had 30-day mortality congruent with that predicted by sPESI and 90-day mortality similar to that from the International Cooperative Pulmonary Embolism Registry. Although there undoubtedly was some selection bias in which patients underwent procedures, the fact that in patients in the PSM group, the mortality was predicted by a validated risk score and was comparable to registry data argues that the selection bias could not alter the predicted mortality.

**Table 6:** Mortality based on select stratification.

	Thrombectomy group				Propensity score-matched group			
	n	30 days	1 year	2 years	n	30 days	1 year	2 years
Unstratified	311	15 (4.8)	40 (12.9)	50 (16.1)	309	28 (9.1)	65 (21)	79 (25.6)
sPESI+	261	14 (5.4)	36 (13.8)	47 (18)	258	26 (10.1)	61 (23.6)	75 (29)
PESI III+	208	14 (6.7)	35 (16.8)	45 (21.6)	201	26 (12.9)	62 (30.8)	75 (37.3)
ESC ILR	42	3 (7.1)	7 (16.7)	8 (19)	179	9 (5)	37 (20.6)	47 (26.3)
ESC IHR	181	5 (2.8)	18 (9.9)	25 (13.8)	59	5 (8.4)	11 (18.6)	13 (22)
ESC HR	39	6 (15.4)	13 (33.3)	15 (38.5)	23	12 (52.2)	14 (60.8)	17 (73.9)
ESC IHR or HR	220	11 (5)	31 (14.1)	34 (15.5)	82	17 (20.7)	25 (30.5)	30 (36.6)
sPESI or PESI III+ no mets	237	10 (4.2)	25 (10.5)	32 (13.5)	215	13 (6)	29 (13.5)	41 (19.1)
ESC IHR or HR no mets	203	8 (3.9)	21 (10.3)	27 (13.3)	76	14 (18.4)	21 (27.6)	26 (34.2)
Massive PE (SBP <90 mm Hg)	35	6 (17.1)	12 (34.3)	12 (34.3)	20	10 (50)	12 (60)	14 (70)
PESI class I or II	103	1 (0.97)	4 (3.8)	5 (4.8)	108	2 (1.8)	2 (1.8)	5 (4.6)
PESI class I or II + RV strain	91	1 (1.1)	4 (4.4)	5 (5.5)	29	0 (0)	0 (0)	1 (3.4)

Values are n (%).

ESC, European Society of Cardiology; HR, high risk; IHR, intermediate high risk; PE, pulmonary embolism; SBP, systolic blood pressure; PESI, pulmonary embolism severity index.

Regarding mortality, there was a significant difference between the thrombectomy and PSM groups which was more pronounced with increasing risk level. However, there were some nuances. Given the higher amount of metastatic cancer in the PSM group, we found that once patients with metastatic cancer were eliminated from the analysis, the mortality benefit only applied to higher-risk patients. This finding further suggests that thrombectomy only provides a mortality benefit for those patients at higher risk.

In patients who were classified into PESI class I or II but had either RV strain or positive cardiac biomarkers or both, we found no significant difference in length of stay, adverse events, or mortality up to 2 years between patients who underwent thrombectomy and those who did. Although there were a limited number of patients included in this comparison, this also signals toward deferral of invasive procedures in patients with clinical stability. This finding also demonstrates the power of the PESI score and why it may continue to have a role in patient selection in the future.

## Conclusion

Within the limitations of a single-center, retrospective review, this study suggests that there is a benefit for pulmonary artery thrombectomy in select patients, particularly in higher-risk patients. The mortality benefit was seen at 30 days with survival curves continuing to separate at 2 years. The results also suggest that PESI class I and II patients with or without right ventricular strain can be safely managed with anticoagulation.

Clinicians should be aware of the differences in the distribution in the length of stay, as well as nonsignificant but increased numerical count of stroke, requirement for transfusion, and recurrent PE in patients undergoing thrombectomy.

Future trials assessing thrombectomy should assess mortality along with long-term functional outcomes and rates of pulmonary hypertension as these are often cited for offering thrombectomy to lower-risk patients. Results of current and past CDT trials that evaluated intermediate high-risk patients should not be extrapolated to lower-risk patients. Another group to be studied in future trials is patients who are classified into PESI class I or II but have RV strain on CT.
